# A New User-Friendly and Affordable Method in Descemet Stripping Endothelial Keratoplasty

**DOI:** 10.2174/1874364101812010242

**Published:** 2018-08-29

**Authors:** Mohammad Soleimani, Seyed Ali Tabatabaei, Reza Mirshahi

**Affiliations:** Eye Research center, Farabi Eye Hospital, Tehran University of Medical Sciences, Tehran, Iran

**Keywords:** DSAEK, Lenticule, Technique, Endothelial keratoplasty, ECD, PBK

## Abstract

**Aims:**

To describe a new technique in Descemet Stripping Automated Endothelial Keratoplasty (DSAEK).

**Materials and Methods:**

In this technique, we use easily available materials (mainly a Nelaton tube) to make an injector for loading DSAEK lenticule and also easily pulling it (using a gauge 23 needle) into the eye. In this paper, we report outcomes of this technique in four cases.

**Results:**

Using these available instruments could easily lead to a clear postoperative cornea. Mean Endothelial Cell Density (ECD) loss at sixth postoperative month was 26%.

**Conclusion:**

We proposed a novel effective user-friendly and affordable technique to perform DSAEK.

## 1 BACKGROUND

Descemet Stripping and Automated Endothelial Keratoplasty (DSAEK) is a selective lamellar corneal graft to replace the impaired endothelium layer [1]. There are numerous indications for performing DSAEK such as Fuchs endothelial dystrophy, Pseudophakic Bullous Keratopathy (PBK) and Iridocorneal Endothelial (ICE) syndrome eliminating the unwanted complications of Penetrating Keratoplasty (PKP) in these circumstances [2].

DSAEK involves dissection of donor’s stroma *via* a microkeratome and inserting the folded posterior lenticule into the patient’s Anterior Chamber (AC) after proper descemet – stripping [3-7]. Following the spreading and popularity of the DSAEK technique, new perspectives of potential complications were revealed including the endothelial trauma to the donor tissue resulting from the manipulation of the lenticule during insertion [7].

In the present study, we aim to describe an inexpensive modification to simplify lenticule delivery and minimize iatrogenic injury in a series of patients.

## 2 CASE PRESENTATION (CASE SERIES)

Herein, we report four consecutive cases of bullous keratopathy undergoing DSAEK procedure Table **1**. Cases 1-3 underwent DSAEK in pseudophakic bullous keratopathy. A combined artisan Intraocular Lens (IOL) implantation and DSAEK was performed for case 4 because of Aphakic Bullous Keratopathy (ABK). Actually, that patient experienced a staphylococcal keratitis in the background of ABK. After administration of fortified cefazolin, his vision was counting fingers at 1meter at the time of scar formation. DSAEK procedure was done two months later.

## 3 INVESTIGATIONS

Preoperative, first and sixth-month central Endothelial Cell Densities (ECDs) were measured using a non-contact specular microscope (TOPCON SP-2000P, Topcon, Tokyo, Japan).

## 4 TREATMENT

After marking superficial cornea with an 8-mm trephination, the endothelial layer is stained *via* trypan blue through a temporal corneal incision. Afterward, a 360^o^ descemetorhexis is fashioned along the trephination mark. Upon preparing the DSAEK lenticule, it is placed unfolded within a Nelaton catheter, cut using a Westcott scissor. It is preferred to create a beveled edge at the tip of the catheter to facilitate the introduction of lenticule into the AC Fig. (**1a**). In the following steps, an AC maintainer is fixed from a vertical corneal incision to provide a formed AC during donor’s tissue delivery. The perpendicular intrastromal channel for the inflow, prevent disturbance of the lenticule by the tip of the instrument. Later, a clear corneal incision is made at nasal side opposite to the 5mm incision. The beveled tip of the catheter is inserted in the AC through the temporal incision and by means of a 23-gauge needle from the opposite nasal port; the lenticule is pulled in to the AC Figs. (**1b** and **1c**). Fig. (**1d**) presents a schematic view of pulling the lenticule into the eye.Finally, an air bubble is injected to the AC to maintain the lenticule position and the temporal incision is closed by 10-0 nylon sutures.

Attached video file (**Video 1**) completely explains this surgical technique.

## 5 OUTCOMES AND FOLLOW-UP

All grafts remain clear after six months. Table **1** shows ECD at different follow-ups, as the table shows mean ECD loss after six months was 26%. We documented an ECD loss of 30% at sixth month in case 4 that underwent combined artisan IOL implantation and DSAEK, however, the cornea was clear with a best corrected visual acuity of 20/30.

## 6 DISCUSSION

In the current study, we introduced a new modification to DSAEK procedure using a device for lenticule delivery which is easily prepared intra-operatively. We believe that using this new technique, the iatrogenic trauma by the forceps during tissue grasping or during taco technique, could be reduced.

Ide *et al.* investigated in vitro damage of the forceps on donor endothelial layer. By means of vital dye, two parallel thick lines of devitalized cells matching the arms of forceps with other scattered areas of injury were identified in those models. Albeit, the crushing damage in the surgical setting can be substantially severe [7].

In 2007, Bradley and McCartney introduced a new technique for lenticule delivery using a double armed 10-0 Prolene suture which was applied on the apex of the donor’s tissue. It was claimed that with suture-dragging, the lenticule positioning is simplified [8]. However, it is debated that further graft damage may ensue including the cheese-wiring of the sutures during dragging and subsequently the need for manipulation by the forceps. In addition, it seems that the surgery is prolonged by suture-drag technique [9].

The pull-through technique discussed by Aralikatti *et al*. or using Busin glide is somehow similar to ours. They used a forcep for the insertion of graft tissue from a glide [9]. Our modification differs regarding the usage of widely available instruments in operation rooms and considering the fact that the small tip of 23-gauge needle is potentially the only surface that touches the lenticule. The decrease in ECD after six months was 26% in our proposed technique that was similar to Busin glide .

## CONCLUSION

In conclusion, we postulate a new easy and affordable method to load and introduce the DSAEK lenticule into the AC. We need more studies to see if iatrogenic injury would be significantly mitigated resulting in better outcomes and longer graft survival.

Take Home Message:

1. DSAEK is a good technique for rehabilitating edematous cornea. Pull-through technique could decrease the risk of iatrogenic trauma to the lenticule.
2. We propose a new easy and affordable technique to load and introduce the DSAEK lenticule into the AC.

## Figures and Tables

**Fig. (1a):**
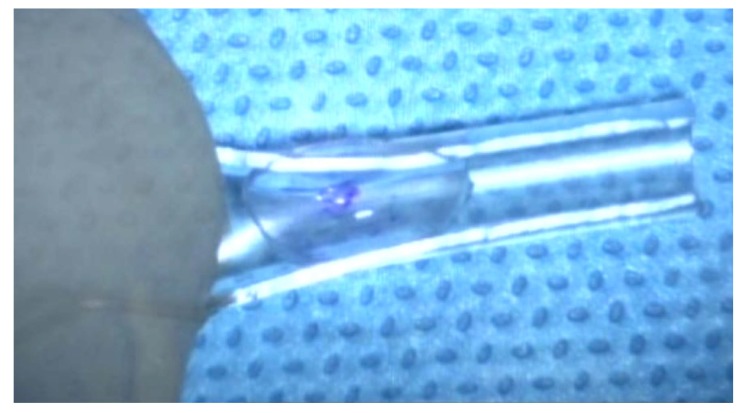
The injector was made of nelaton tube, the lenticule was loaded in the injector.

**Fig. (1b):**
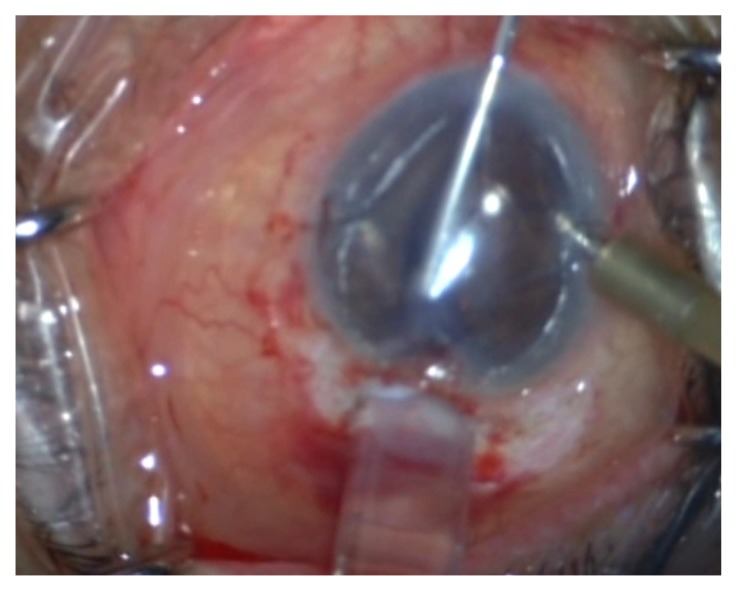
Pulling the lenticule using a gauge 23 needle.

**Fig. (1c):**
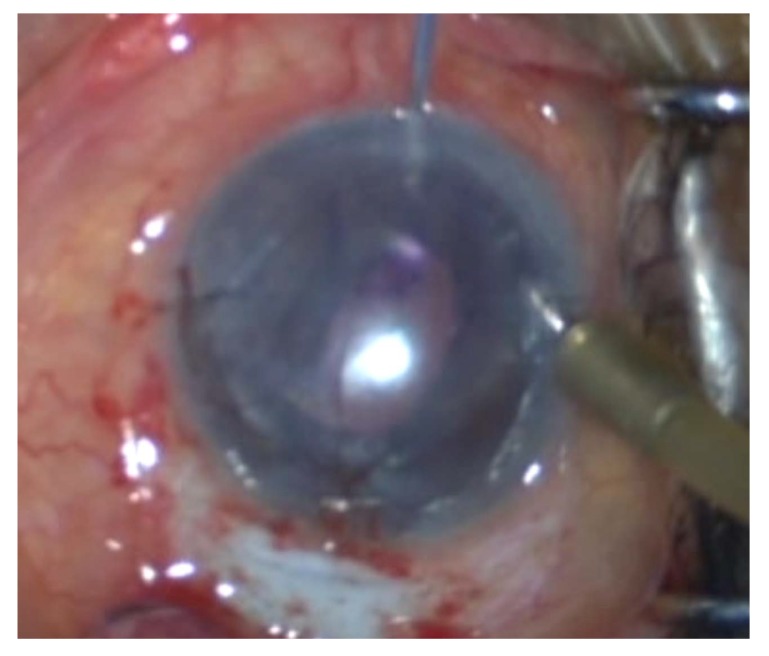
Pulling the lenticule using a gauge 23 needle.

**Fig. (1d):**
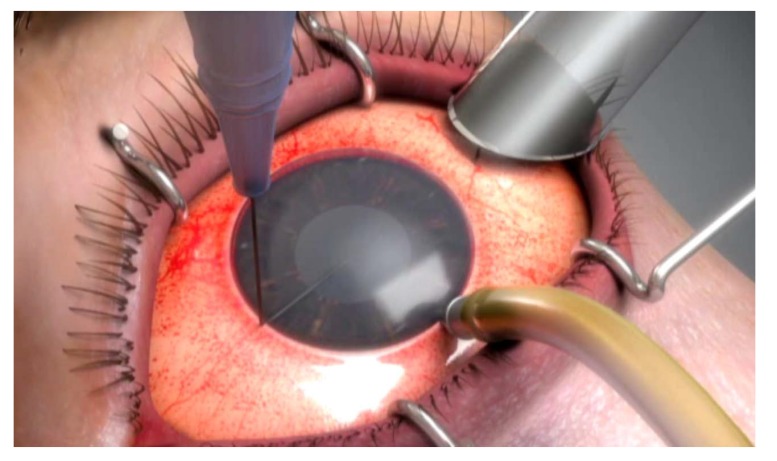
A schematic view of pulling the lenticule into the eye.

**Table 1:** Pre-operative Endothelial Cell Density in the Donor and First and Six Month Values. ECD: Endothelial Cell Density (n/mm2).

Patient	Preoperative ECD	First post-operative month ECD	Sixth post-operative month ECD	Decrease in ECD
1	2834	2022	2102	26%
2	3345	2745	2704	19%
3	2920	2253	2145	27%
4	3053	2083	2025	30%
